# Engineering the Cellular Microenvironment for Human Induced Pluripotent Stem Cell Cardiac Differentiation: Beyond Wnt Signaling

**DOI:** 10.3390/bioengineering13091062

**Published:** 2026-09-12

**Authors:** Gustavo Rosero, Ana Belén Peñaherrera-Pazmiño, Camilo Pérez-Sosa

**Affiliations:** 1 Carrera de Ingeniería Civil, Mecánica Computacional e Inteligencia Artificial Aplicada (MCIAA), Facultad de Ciencias de La Ingeniería e Industrias, Universidad UTE, Quito 170527, Ecuador; 2 Centro de Investigación Biomédica (CENBIO), Facultad de Ciencias de La Salud Eugenio Espejo, Universidad UTE, Quito 170527, Ecuador

**Keywords:** human induced pluripotent stem cells (hiPSCs), cardiomyocyte maturation, Wnt/β-catenin signaling, cardiac developmental niche, tissue engineering, hydrogels, heart-on-chip, regenerative medicine, SDG 3

## Abstract

Human induced pluripotent stem cells (hiPSCs) have revolutionized cardiovascular research by providing a renewable source of patient-specific cardiomyocytes for disease modeling, drug discovery, precision medicine, and regenerative therapies. Temporal modulation of canonical Wnt/β-catenin signaling has established the current gold standard for efficient and reproducible cardiac differentiation under chemically defined conditions. However, conventional Wnt-based protocols consistently generate cardiomyocytes with fetal-like structural, electrophysiological, metabolic, and contractile characteristics, highlighting that lineage specification alone is insufficient to achieve functional maturation. This review discusses recent advances in engineering the cardiac developmental niche by integrating extracellular matrix remodeling, biomaterials, biomechanical and bioelectrical stimulation, metabolic regulation, multicellular interactions, and microfluidic technologies to better recapitulate the dynamic microenvironment of human cardiogenesis. We further examine how emerging bioengineered platforms, including engineered heart tissues, cardiac organoids, and heart-on-chip systems, enhance the physiological relevance of hiPSC-derived cardiac models. Finally, we discuss future perspectives arising from the convergence of developmental biology, tissue engineering, biomaterials, artificial intelligence, and microphysiological systems, proposing that the next generation of cardiac differentiation platforms will depend on integrating canonical Wnt signaling within biomimetic developmental microenvironments to generate mature human cardiac tissues with enhanced translational potential. By advancing physiologically relevant human cardiac models for disease modeling, drug discovery, and regenerative medicine, this work also supports the research and innovation priorities underlying Sustainable Development Goal 3 (SDG 3), particularly those related to reducing the burden of non-communicable diseases and strengthening health-related research and development.

## 1. Introduction

Cardiovascular diseases remain a leading cause of morbidity and mortality worldwide, while the study of human cardiac biology continues to be constrained by the limited availability of adult human cardiac tissue and the incomplete translational relevance of conventional animal and immortalized cell models. Human induced pluripotent stem cells (hiPSCs) have addressed these limitations by providing a renewable and patient-specific source of cardiomyocytes capable of recapitulating key features of human cardiac development, physiology, and disease. In contrast to conventional animal models and immortalized cell lines, hiPSC-derived cardiomyocytes (hiPSC-CMs) enable the investigation of patient-specific phenotypes, disease mechanisms, drug-induced cardiotoxicity, and regenerative strategies within a human genetic background. Continuous advances in cardiac differentiation and tissue engineering have further expanded the utility of hiPSC-based models from disease modeling toward precision medicine, drug discovery, and the development of increasingly sophisticated human cardiac tissues. Consequently, hiPSC technology has become an important platform for studying both normal cardiogenesis and pathological cardiac remodeling, while providing a foundation for developing more physiologically relevant in vitro cardiac models [1,2,3,4,5,6].

The development of chemically defined cardiac differentiation protocols based on temporal modulation of canonical Wnt/β-catenin signaling has substantially improved the efficiency, reproducibility, and scalability of cardiomyocyte generation from human pluripotent stem cells. By recapitulating the sequential activation and inhibition of Wnt signaling observed during early cardiogenesis, these approaches provide a robust framework for directing mesoderm formation and subsequent cardiac lineage specification under defined culture conditions. Although this strategy has become a central component of contemporary cardiac differentiation protocols, increasing evidence indicates that efficient control of lineage specification alone does not reproduce the full developmental complexity required for cardiomyocyte maturation. Thus, while Wnt modulation provides a critical molecular foundation for cardiac differentiation, the structural and functional properties of the resulting cells remain strongly influenced by the cellular and physicochemical microenvironment in which differentiation occurs [7,8].

Despite the high efficiency and reproducibility achieved with contemporary cardiac differentiation protocols, hiPSC-derived cardiomyocytes generally retain an immature phenotype that more closely resembles the fetal than the adult myocardium. Importantly, differentiation efficiency and cardiomyocyte purity do not necessarily translate into functional maturation. Conventional hiPSC-CMs frequently display underdeveloped sarcomeric organization, immature calcium handling, limited mitochondrial oxidative capacity, altered electrophysiological properties, and reduced contractile performance compared with adult cardiomyocytes. These limitations represent distinct dimensions of maturation that include structural, electrophysiological, metabolic, and functional/mechanical phenotypes, and their incomplete development remains a major barrier to the physiological relevance and translational utility of hiPSC-derived cardiac models. Increasing evidence therefore suggests that improving cardiomyocyte maturation requires approaches that extend beyond efficient lineage specification and address the developmental context in which cardiac cells acquire their mature phenotype [9,10].

In this context, the cardiac developmental microenvironment should be considered as an integrated regulatory niche in which biochemical, biomechanical, bioelectrical, metabolic, and multicellular cues interact with developmental signaling to shape cardiomyocyte fate and maturation [11,12]. Rather than focusing on individual maturation strategies in isolation, this review examines how these complementary cues can be integrated with canonical Wnt signaling across cardiac differentiation and maturation, with emphasis on four major maturation outcomes: structural, electrophysiological, metabolic, and functional/mechanical phenotypes [10,13,14]. By linking developmental signaling with microenvironmental engineering, we aim to provide a critical framework for comparing current 2D and 3D platforms and identifying the principal challenges and opportunities for reproducible, scalable, and physiologically relevant cardiac tissue models.

Scope of the review. This review focuses on the integration of canonical Wnt signaling with engineered cardiac microenvironments to improve the maturation of human pluripotent stem cell-derived cardiac models. Rather than providing a comprehensive developmental atlas of all cardiac cell lineages or a systematic review of individual bioengineering technologies, we examine how biochemical, extracellular matrix, biomechanical, bioelectrical, metabolic, and multicellular cues can complement temporally controlled Wnt modulation during cardiac differentiation and maturation. Particular emphasis is placed on human iPSC-based systems and on the transition from conventional two-dimensional cultures toward increasingly complex three-dimensional and bioengineered cardiac platforms, including spheroids, organoids, heart-on-chip systems, and engineered heart tissues. This framework is intended to identify how microenvironmental engineering can bridge the gap between efficient lineage specification and physiologically relevant cardiac maturation.

## 2. Wnt Signaling as the Cornerstone of Cardiac Differentiation

Canonical Wnt/β-catenin signaling plays a biphasic and tightly regulated role during cardiac development, acting as a major determinant of mesoderm induction and subsequent cardiac lineage specification. During early gastrulation, transient Wnt activation promotes primitive streak and mesoderm formation through the induction of developmental regulators such as Brachyury (T) and MESP1, thereby establishing cardiovascular progenitor identity. As development progresses, however, sustained Wnt activity becomes inhibitory to cardiogenesis and must be appropriately attenuated to permit commitment toward the cardiac lineage and activation of cardiac transcription factors, including NKX2-5, GATA4, TBX5, and MEF2C. Importantly, this developmental switch is governed not simply by pathway activation or inhibition, but by the precise timing, duration, and magnitude of Wnt signaling within a changing cellular context. Consequently, differences in the temporal window and intensity of Wnt modulation can influence both the efficiency of cardiac specification and the phenotype of the resulting cardiomyocytes, providing the mechanistic basis for contemporary human pluripotent stem cell differentiation strategies [15,16].

Importantly, the developmental role of Wnt signaling extends beyond cardiomyocyte specification and is strongly dependent on developmental stage and cellular context. During cardiac development, temporally coordinated Wnt activity contributes not only to mesoderm and cardiogenic progenitor formation, but also to the development of the second heart field, endocardial and valvular structures, epicardium, and coronary vasculature. Human pluripotent stem cell-based models further support the importance of temporal pathway regulation for directing distinct cardiac developmental programs, emphasizing that Wnt activity must be interpreted within a broader spatiotemporal and multicellular context [3,17]. These observations reinforce the concept that Wnt modulation provides a developmental framework for cardiac lineage specification, whereas appropriate extracellular, mechanical, metabolic, and multicellular cues are required to reproduce the coordinated maturation of cardiac tissues.

The recognition of this biphasic signaling behavior has driven the development of chemically defined differentiation protocols that use temporally controlled small-molecule modulation to recapitulate key stages of embryonic cardiogenesis. Transient activation of canonical Wnt signaling, most commonly through glycogen synthase kinase-3 (GSK-3) inhibition with CHIR99021, promotes mesoderm induction, whereas subsequent inhibition of Wnt secretion or β-catenin activity using compounds such as IWP2, IWR-1, or Wnt-C59 favors cardiac lineage specification. Although these protocols have markedly improved reproducibility, scalability, and cardiomyocyte yield, their outcomes remain sensitive to treatment timing, compound concentration, cell density, and cell-line-specific responses. Importantly, Wnt signaling does not operate in isolation; complementary intracellular pathways, including PI3K/AKT/mTOR signaling, contribute to cardiac cell survival, growth, metabolism, and remodeling, further emphasizing the networked nature of cardiac signaling [18]. Thus, the biological outcome of Wnt modulation depends on the integration of pathway activity with the broader intracellular and extracellular context rather than on Wnt activity alone [17,19].

The implementation of temporally controlled Wnt modulation represented a major advance in cardiac differentiation by replacing poorly defined culture conditions with reproducible, chemically defined protocols applicable across multiple human pluripotent stem cell lines. These strategies can consistently achieve high cardiomyocyte yields, often exceeding 80–90% under optimized conditions, without genetic manipulation or cell sorting, thereby supporting scalable applications in disease modeling and drug screening. However, differentiation efficiency or cardiomyocyte purity alone does not constitute evidence of functional maturation. Cardiomyocytes generated through conventional Wnt-based protocols frequently retain fetal-like structural, electrophysiological, metabolic, and contractile features, indicating that robust lineage specification can occur without complete acquisition of adult-like properties. Importantly, experimental studies have shown that relatively subtle changes in the timing and duration of Wnt activation and inhibition can influence not only cardiac lineage commitment but also subsequent maturation, highlighting the importance of developmental timing when optimizing differentiation protocols. Thus, maximizing cardiomyocyte yield should not be considered an endpoint in itself; rather, differentiation conditions should be evaluated according to the structural, electrophysiological, metabolic, and functional properties of the resulting cells [13,14].

Although temporal modulation of canonical Wnt signaling provides a robust framework for cardiac lineage specification, developmental signaling alone cannot fully reproduce the complexity of human cardiogenesis. During embryonic heart development, Wnt activity is embedded within a dynamic microenvironment in which extracellular matrix composition, mechanical forces, cell–cell interactions, oxygen availability, and metabolic transitions continuously shape cellular responses and tissue maturation. These environmental cues do not act independently of intracellular signaling; rather, they can modulate how developmental pathways are interpreted by regulating cell adhesion, mechanotransduction, cytoskeletal organization, and metabolic state. Consequently, the same Wnt-directed differentiation protocol may produce different phenotypic outcomes depending on the surrounding cellular and physicochemical context. This relationship provides a rationale for integrating canonical Wnt modulation with engineered microenvironmental cues, shifting cardiac differentiation from the optimization of lineage specification alone toward the coordinated control of developmental signaling and tissue-level maturation [11,12].

**Figure 1:**
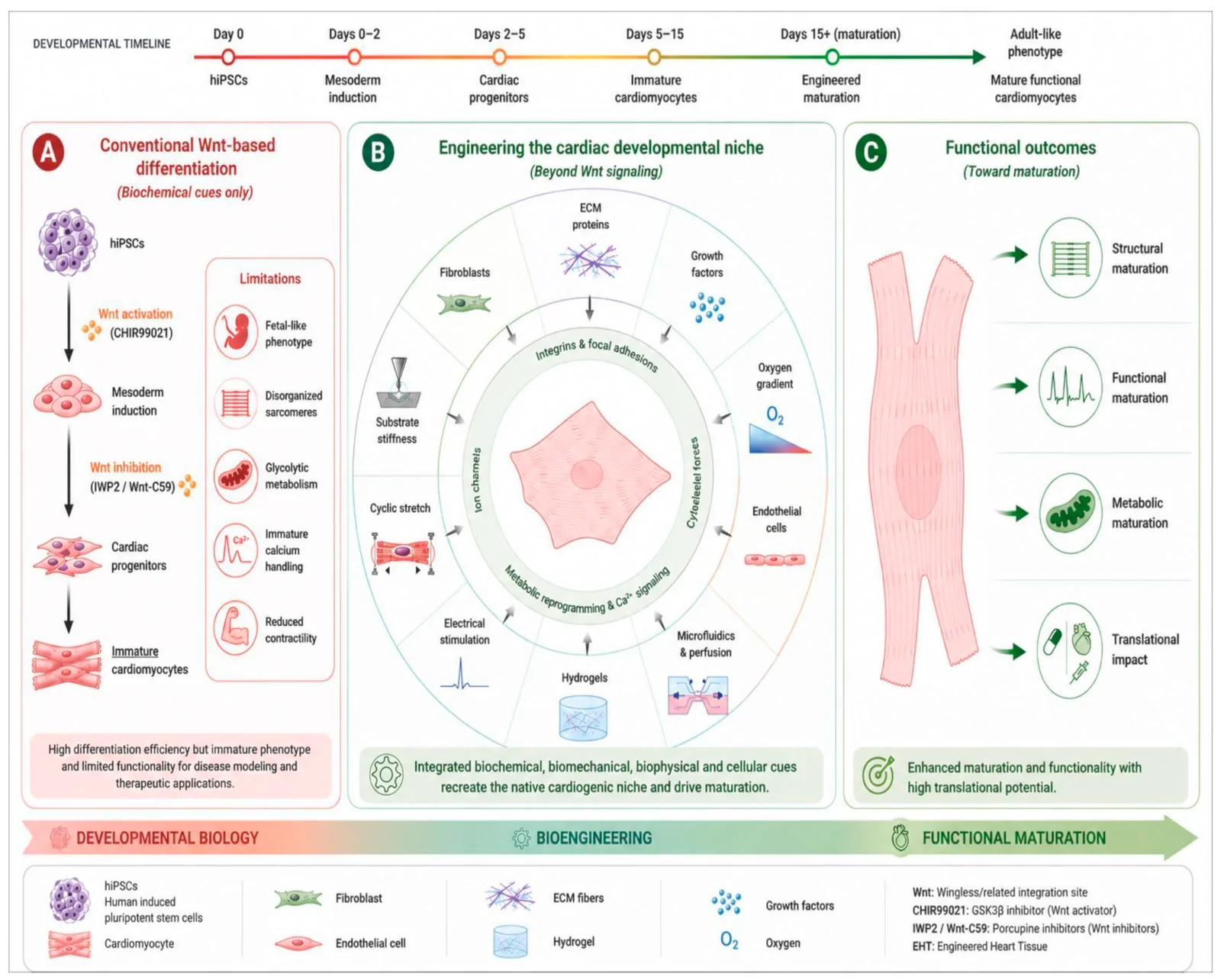
Engineering the cardiac developmental niche beyond canonical Wnt signaling promotes functional maturation of human pluripotent stem cell-derived cardiomyocytes. Schematic representation of the transition from conventional Wnt-directed cardiac differentiation to bioengineered developmental microenvironments that recapitulate key biochemical, biomechanical, biophysical, and multicellular cues of native cardiogenesis. While temporal modulation of canonical Wnt signaling efficiently specifies the cardiac lineage, faithful reconstruction of the developmental niche through extracellular matrix remodeling, mechanical stimulation, oxygen regulation, soluble factors, multicellular interactions, hydrogels, and microfluidic perfusion promotes progressive structural, metabolic, electrophysiological, and functional maturation. Collectively, these integrated engineering strategies drive the generation of physiologically relevant human cardiac tissues with enhanced translational potential for disease modeling, drug discovery, and regenerative medicine.

Taken together, these considerations support a shift from differentiation protocols optimized primarily for cardiac lineage specification toward integrated strategies that recreate key features of the cardiac developmental niche. Figure 1 summarizes this conceptual transition, illustrating how temporal Wnt modulation can be complemented by biochemical, biomechanical, bioelectrical, metabolic, and multicellular cues to progressively enhance the structural and functional maturation of hiPSC-derived cardiomyocytes. This framework provides the basis for the bioengineering strategies discussed in the following sections, where individual microenvironmental components and their integration into two- and three-dimensional cardiac platforms are critically examined.

## 3. Engineering the Microenvironment During Cardiac Differentiation

### 3.1. Extracellular Matrix

Interactions between cardiac cells, neighboring cells, and their extracellular surroundings are essential for heart function across scales, ranging from single cells to the entire organ. The cardiac extracellular matrix offers structural support while also actively controlling cell behavior through continuously perceived and interpreted mechanical and biochemical cues. Cardiomyocytes attach to extracellular matrix components—such as collagens, laminins, and fibronectin—via dedicated adhesion complexes, thereby linking contractile activity with the surrounding matrix [20].

A major factor in effective cardiomyocyte differentiation is the extracellular matrix (ECM), which not only offers structural support but also governs cell adhesion, survival, and lineage determination via integrin-mediated signaling [21]. In fact, an essential role of fibronectin in promoting the initial stages of human pluripotent stem cell cardiac differentiation has been reported. It acts via transmembrane integrin α4β1 and αVβ1 receptors to activate downstream integrin-linked kinase signaling cascades and increase pAKT levels, thereby promoting formation of Brachyury-positive mesoderm (Table 1) [22].

To faithfully recapitulate the native cardiac microenvironment, the synergistic interplay of fibronectin and Matrigel composite in cardiomyocyte differentiation has been explored. This engineered microenvironment integrates fibronectin-driven integrin engagement and focal adhesion signaling with the structural and biochemical backing provided by Matrigel’s basement membrane-like matrix, thereby forming a dual-modality extracellular matrix (ECM) niche to reflect the native cardiac milieu (Table 3). Cells cultured on the composite showed greater expression of key cardiac transcription factors (NKX2.5) and sarcomeric proteins (TNNT2/cTnT) and structural proteins (MYH6, cTnT), in addition to enhanced contractile performance and a more developed sarcomeric organization. Notably, the differentiated cardiomyocytes showed spontaneous contractility and responsiveness to pharmacological agents (verapamil and isoproterenol) compared with single-component ECMs [21].

### 3.2. Hydrogels and Biomaterials

There are two broad classes of hydrogels: natural and synthetic. Natural hydrogels are highly biocompatible and have a higher affinity for cells. In contrast, they have weaker mechanical strength and degrade at a faster rate than synthetic polymers. Although synthetic polymers offer stronger mechanical properties than natural ones, they may not be as biocompatible [23]. To harness the strengths of both, hybrid hydrogels have been created. Among these novel hybrid hydrogels, the incorporation of conductive nanomaterials, like carbon nanotubes (CNTs) [24], graphene oxide [25], and gold (Au) nanoparticles integrated into the chitosan-silk fibroin hydrogel (Au@Ch-SF) [26] has been proposed. For instance, polydopamine-reduced graphene oxide was incorporated into gelatin methacrylate (GelMA) hydrogels (GelMA-PDA-rGO), and an increase in cell survival rate and faster maturation was reported compared to the GelMA hydrogel alone [23].

**Figure 2:**
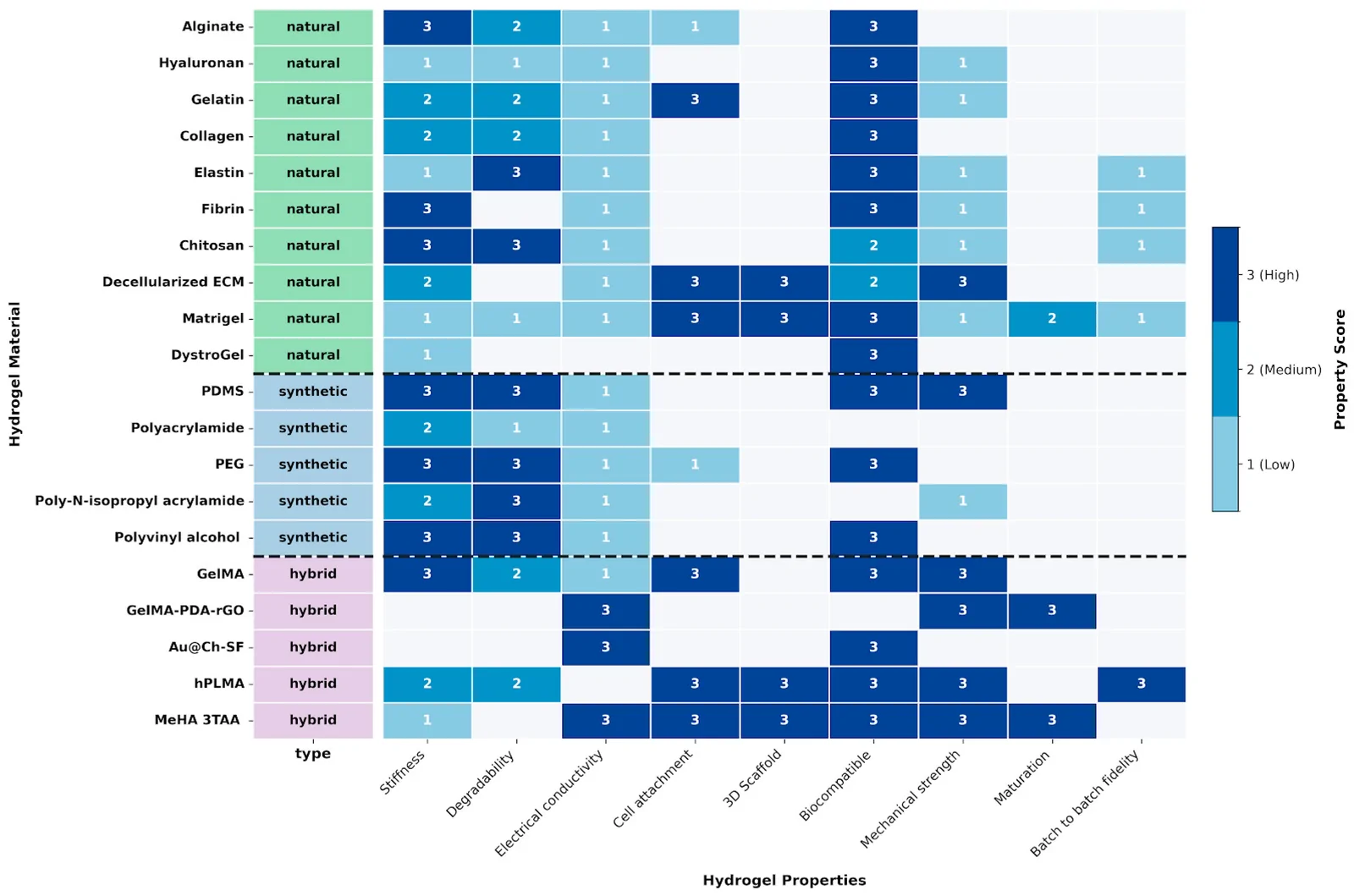
Scored hydrogel properties for cardiac tissue engineering. The first column indicates the hydrogel type. Light blue represents low property values; medium blue denotes intermediate property values, and deep navy blue highlights optimal/high property values. (e.g., lower stiffness of 1–10 kPa, medium stiffness 11–19 kPa, high stiffness ≥ 20). (e.g., short matrix persistence over days, intermediate persistence over weeks, and long persistence over months) [23,24,25,26,27,28,29,30,31,32,33,34,35,36,37,38,39,40,41]. Scores represent a semi-quantitative synthesis of reported material properties and are intended for comparative visualization rather than quantitative meta-analysis.

Figure 2 summarizes selected hydrogel properties relevant to cardiac tissue engineering, highlighting their potential advantages and limitations. An ideal scaffold should be biocompatible, have enough mechanical strength and electrical conductance to perform dynamic functions of the heart [27].

Matrigel comprises numerous proteins present in the ECM such as collagen type IV, perlecan, laminin, and entactin alongside other constituents [42,43,44,45]. It has been the gold standard for 3D cell culture for half a century. Although it can reproduce several biochemical features of basement-membrane ECM, its tumor-derived origin, undefined composition, and batch variability limit reproducibility and translational standardization. Moreover, Matrigel’s complex and undefined composition causes high batch-to-batch variability and low reproducibility, which complicates result interpretation [45,46]. These limitations restrict Matrigel’s utility for standardized and translational cardiac tissue engineering. Therefore, there is a need to develop natural and synthetic biomaterials to serve as alternative substrates for cell culture, proliferation and differentiation [23]. Consequently, other sources have been explored, and human-derived materials such as platelet lysates (hPL), which possess albumin, fibronectin, and growth factors that enhance cell proliferation [45]. However, hPL lacks intrinsic mechanical properties. To address this issue, a commercially available formulation of hPL is methacryloyl human platelet lysates (hPLMA, Metatissue^®^, Ílhavo, Portugal), which have been shown to improve the mechanical robustness and processability of hPL-based materials. Indeed, hPLMA hydrogels at 15% (*w*/*v*) have a Young’s modulus ranging from 15 to 20 kPa. In contrast, Matrigel hydrogels at 1.2% (*w*/*v*) have a significantly lower Young’s modulus of 450 Pa. Furthermore, stiffness and porosity can be regulated by modulating the degree of methacrylation and hPLMA concentration [47]. hPLMA composition includes vitronectin, a human adhesion protein that promotes integrin-mediated attachment. In the realm of long-term cell culture, Matrigel degrades within approximately 7 days, whereas hPLMA has supported sustained cell growth for up to 14 days. Building on these advantages, hPLMA provides a robust and reproducible human-derived alternative to Matrigel as it is an ethically sustainable platform for next-generation 3D cell culture and tissue engineering applications [48].

In the context of biomaterials, microfibrous topographical cues together with surface nano-topographic patterns smaller than 100 nm not only facilitate appropriate cardiomyocyte adhesion but also replicate the native architecture of cardiac tissue [49]. Notably, the cardiac ECM modulates cellular growth and provides a supporting environment for cardiac cells such as cardiomyocytes, vascular cells, fibroblasts, and inflammatory cells, which contribute to the synthesis and degradation of ECM proteins in and around the cardiac ECM [50]. Moreover, ECM stiffness regulates the ability of CMs to divide and mature. As the heart develops, the levels of collagen I, collagen III, and laminin progressively increase, whereas fibronectin and periostin levels decrease with age. This suggests that ECM components change throughout development and may modulate the regenerative capacity of CMs [51,52]. The importance of ECM in heart development has been strengthened by the generation of 3D mouse heart organoids in vitro by incorporating the laminin–entactin complex and exogenous fibroblast growth factor 4. These organoids formed from self-organizing embryoid bodies, resulting in atrium- and ventricle-like contractile structures [53].

Although human embryonic stem cell-derived cardiomyocyte (hESC-CMs) transplants have significantly advanced heart regeneration research in preclinical models, post-implantation cell survival remains exceptionally poor. This low retention rate occurs because transplanting the cells individually triggers anoikis. Therefore, Lee et al. 2020 [53] discovered that the combination of hESC-CMs and hyaluronate-based hydrogel was the best in improving cardiac functional recovery, delaying left ventricular remodelling and preventing arrhythmias in rat acute myocardial infarction models. This could be attributed to hyaluronate’s rapid degradation in vivo within 12 h after injection, making it less likely to cause inflammatory responses within the heart.

Despite extensive research in the biofabrication of pathophysiological environments, these systems often lack disease-specific ECM features. To mitigate these challenges, DystroGel, a hydrogel derived from the cardiac ECM of a porcine Duchenne muscular dystrophy model, has been utilized to demonstrate the importance of applying a 3D substrate that reflects intercellular dynamics, as it recapitulates the specific molecular composition of dystrophic cardiac tissue [54].

To achieve such precise, disease-specific tissue recapitulation, advanced fabrication strategies are required. Bioprinting is a process of additive manufacturing that creates two-dimensional (2D) or three-dimensional (3D) biological objects. The development of bioprinting technology originated with the adaptation of commercial inkjet and laser-based platforms to deposit cell suspensions and low-viscosity cell–matrix hydrogels in 2D configurations. Subsequently, 3D construct fabrication was achieved through the incremental elevation of the printhead along the z-axis [55]. Besides creating 2D and 3D structures from biomaterials, this method enables the integration of extracellular matrix components, proteins, growth factors, other biochemical agents, and live cells into the layers [55].

Cardiovascular tissues comprise a highly organized and complex 3D structure that makes them challenging to fabricate in a biomimetic manner by conventional scaffold fabrication methods [56]. Over the last decade, instrumented cardiac microphysiological devices have been fabricated via multimaterial 3D printing. Lind et al. 2017 designed functional inks based on piezoresistive, high-conductance, and biocompatible soft materials that facilitate integration of soft strain gauge sensors [57].

3D bioprinting has emerged as a new, cell-based technique because it can reproduce key aspects of biological constructs, including cell density, multicellular structure, the physicochemical environment, and vascularization, using accurately designed architectures [56]. Advances in 3D in vitro platforms have addressed many of the drawbacks of more traditional 2D cell cultures, which lack the adequate mechanical signals and complexity observed in 3D tissues [58,59]. Recently, a single-step digital light processing (DLP) 3D printing strategy was established to rapidly fabricate mold substrates within minutes, bypassing conventional multi-stage processing workflows. These DLP-derived molds facilitated the regulated ECM remodeling driven by human induced pluripotent stem cell-derived cardiomyocytes (iPSC-CMs), yielding engineered cardiac tissues with high batch-to-batch functional fidelity and high reproducibility (>95%) [58]. Furthermore, this model demonstrated improved metabolic and cytoskeletal maturity over 2D models and is useful for modeling pathological cellular processes related to heart disease [60]. Given the inherent structural and compositional complexity of cardiac tissue, cardiac biofabrication requires attention to several key components, namely: (i) the use of cell sources that possess high regenerative capacity, (ii) the selection of biomaterials that can reproduce the native cardiac microenvironment by directing cell fate, offering mechanical stabilization, and remaining non-immunogenic, (iii) a clearly defined structural organization, and (iv) the implementation of relevant physiological stimuli—both mechanical and electrical—to support efficient cellular maturation and functional performance.

Biomaterials derived from the ECM offer an exciting avenue for healing damaged heart tissue by supplying essential biological and physical signals. However, translating preclinical success to human medicine remains in its infancy. Overcoming translational hurdles—such as batch variability and post-implantation immune reactions—through strict manufacturing controls and immunosuppressive strategies is critical to achieving reliable, long-term cardiac regeneration.

### 3.3. Mechanical Properties

Fluid shear stress can influence cell alignment. For example, in the 3D heart-on-a-chip model, the endothelial layer was cultured after continuous perfusion for 4 days, and the human umbilical vein endothelial cells (HUVECs) exhibited parallel alignment to the direction of medium flow. Furthermore, these cells displayed an elongated, spindle shape with a clear directional preference [61]. Within our bodies, vascular endothelial cells are continuously subjected to blood shear stress, which triggers intracellular signaling pathways that subsequently alter cellular behavior. The same study also observed a clear orientation of flow-induced F-actin and the expression of CD31 cell–cell junction protein, which is upregulated by shear stress induced by flow (Table 3). Building on this aspect, the effect of controlled perfusion conditions on developmental and functional maturation of 3D cardiac ventricular microtissues has been evaluated. In fact, tissues cultured with higher flow rates resulted in enhanced structural and functional maturation. In addition, perfusion was related to the upregulation of a wide group of genes with a key role in cardiomyocyte physiology, suggesting enhanced microtissue maturation and contractile performance at the fifth day of culture [62]. Another study assessed the influence of shape recovery by using a shape memory polyurethane in 4D scaffolds. They demonstrated that the mechanical stimulus imparted by shape recovery is able to influence the shape of cells and nuclei [63]. Moreover, a 4D hydrogel-based cardiac patch was constructed with a specific smart design for physiological adaptability using a beam-scanning stereolithography (SL) printing technique. This smart patch provides mechanical support, a physiologically tunable structure, and a suitable matrix environment for cell implantation [64].

In the realm of structural guidance, in vitro, anisotropic structural guidance cues can be replicated through parallel micro-grooves or by using aligned scaffold fibers. Cardiomyocytes are able to sense these topographical cues and orient themselves to the given anisotropy, a process referred to as contact guidance [49,65]. Therefore, culturing cardiomyocytes by integrating topographical cues represents a robust approach for generating a population of mature cardiomyocytes suitable for in vitro disease modeling and other applications. Topographical cues can foster the attachment and elongation of cardiomyocytes in vitro. Such alignment is key for propagating electrical signals and generating the mechanical force required for effective pumping action [49].

In the context of effective in vitro models, they must replicate the intercellular relationships and physicochemical features of native ECM to fully recapitulate disease-specific characteristics. For instance, the myocardium comprises spatially nonuniform mechanical conditions that enhance regional performance. The whole heart is characterized by an anisotropic cardiac tissue structure with a global stiffness (5–30 kPa) [66]. In healthy adults, ventricular myocardial tissue shows elastic moduli between 10–15 kPa; by contrast, fibrotic or infarcted myocardium often increases beyond 20–50 kPa [20,54]. To achieve a Young’s modulus of approximately 20 kPa, fibrinogen was added to a decellularized porcine cardiac matrix to improve the cellular niche for cardiomyocyte cell culture and to avoid the cellular toxicity caused by transglutaminase crosslinking. They used thrombin for fibrin gel formation [20,33,54,66].

In addition to evaluating topographical cues, the synergy between physical cues and biochemical signals is crucial for guiding cardiomyocyte maturation and the formation of functional myocardium [49]. In addition, biomaterials necessitate improvement due to their limited or negligible electrical conductivity [40]. CNT-incorporated photo-cross-linkable GelMA hydrogels have been proposed to create cardiac constructs that provide excellent mechanical integrity and advance electrophysiological functions [24]. In addition, a novel, electrically conductive, photocurable, and 3D-printable hydrogel composed of metacrylated hyaluronic acid (MeHA) conjugated with 3-thiopheneacetic acid (3TAA) has demonstrated biocompatibility due to sustained cardiac and vascular cell activity (Table 2) [40].

### 3.4. Biochemical and Metabolic Cues

Biochemical and biophysical properties guide cardiac tissue morphogenesis [33]. Ideally, a decellularized tissue provides the native cell niche with many of the structural and biochemical cues that naturally interact with the cells of that particular tissue. Although synthetic systems can reproduce selected biochemical and mechanical features, they do not yet fully recapitulate the complexity of tissue-specific microenvironments. Therefore, decellularized myocardial extracellular matrix, dECM, has been proposed as it potentiates cardiomyocyte differentiation and maturation in human embryonic stem cells and rat neonatal ventricular cardiomyocytes [67]. Particularly, porcine cardiac dECM hydrogels are structurally similar to their human analogs [68]. Indeed, it is thought that partial preservation of organ-specific cues, improved by growth factors composed in the dECM, contributes to organ-specific cellular differentiation. As demonstrated by Navaee et al. 2023 [33], the dECM-fibrin hydrogel in conjunction with fibroblast co-culture facilitated the differentiation capacity of H9c2 myoblasts towards a cardiomyocyte phenotype, showing higher frequency, synchronicity, and earlier beating in dECM-fibrin hydrogel. Building on these observations, a dual-modality ECM niche that comprises fibronectin and Matrigel has been demonstrated to activate FAK, ERK, and AKT pathways, promoting lineage-specific commitment and functional maturation [21]. To further provide biochemical modulation, a stage-specific small-molecule-directed differentiation protocol was applied. They determined a 12 μM CHIR99021 concentration for differentiation of pluripotent stem cells into cardiomyocytes. The second small molecule involved was IWP2, which was used at 5 μM on day 3 of the differentiation process [21]. This modulation of Wnt/β-catenin signaling, together with this matrix, synergistically promoted efficient cardiomyocyte specification, sped up sarcomere assembly, and enhanced both the onset and robustness of spontaneous contractile activity.

Currently, cell therapy products are developed in standard systems like well plates and flasks. However, these culture methods are not able to precisely control the cellular microenvironment and cell-to-cell signaling because of their large surface area and volume [69]. In sharp contrast, microfluidics facilitates the accurate control of the cellular microenvironment by reducing the culture volume, making it easier to handle, enhancing cost-effectiveness, and having the potential for automation and parallelization [70]. This confinement influences cellular proliferation and differentiation [71]. In a confined chamber, the intercellular factors and signaling molecules are concentrated; therefore, cellular uptake increases [72]. In this sense, the signal molecules and factors released in the media by cells undergoing reprogramming and differentiation accumulate in the extracellular space, serving as cues for the other surrounding cells to start cellular processes like proliferation and differentiation [70]. Indeed, microfluidic devices enable a tight balance between the addition of exogenous molecules and the accumulation of autocrine cues to lead cell differentiation while maintaining their capacity for self-regulation [73,74]. Building on confined microenvironments, differentiation inside droplets enhances molecular diffusion dynamics. Therefore, it promotes proximity-based signaling [74], and it may further contribute through contact-mediated or surface-dependent biochemical cues [75]. Notably, nitric oxide, fatty acids, and extracellular vesicles are among the signaling mediators involved in endothelial–cardiomyocyte communication and can influence cardiac development and postnatal function [61,76].

**Table 2:** Representative studies comparing bioengineering strategies for maturation of human hiPSC-derived cardiac tissues.

Representative Study	Cell/Model	Engineering Strategy	Key Maturation Outcomes	Main Limitation	Translational Considerations
[22]	hiPSC-derived cardiomyocytes; defined ECM culture	Defined ECM composition, particularly fibronectin	Structural/lineage: enhanced Brachyury^+^ mesoderm and subsequent hPSC-CM generation; FN-dependent integrin/ILK/AKT signaling.	Primarily evaluates ECM-dependent differentiation rather than full adult-like functional maturation.	Moderate: defined ECM improves reproducibility and reduces reliance on poorly defined matrices.
[77]	hiPSC-derived cardiac spheroids containing CMs, ECs, SMCs and CFs	Multicellular scaffold-free 3D spheroids	Structural/electrophysiological/metabolic: improved sarcomere maturation; higher HCN4 and N-cadherin; altered CM bioenergetics; conduction velocity in isolated C4 CMs ~59.3 ± 14.7 cm/s	Long culture period and lack of vascular perfusion; maturation improvements were not uniform across endpoints	Moderate–high: relatively simple and potentially scalable multicellular platform
[40]	hiPSC-derived cardiomyocytes; defined ECM culture	Printable conductive biopolymer. Multifunctional hydrogel composed of MeHA conjugated with 3TAA. 3D printing	Improvement in conductivity achieved through the chemical conjugation of MeHA and 3TAA (2499 μS). The beat rate (80 bpm) of the hiPSC-derived cardiomyocytes encapsulated in these hydrogels was calculated by using the average displacement over time (24 μm).		Moderate: hemocompatibility tests in accordance with regulatory guidelines and standards will be required for preclinical in vivo studies and clinical translation.
[78]	hiPSC-CM engineered heart tissues	Controlled mechanical afterload	Structural/functional: sarcomere and cell elongation, improved Ca^2+^ handling and adult MYH isoform expression; optimal functional response at intermediate loading (~0.45)	Excessive loading induced hypertrophy/fibrosis-associated responses	High mechanistic value; moderate scalability: adjustable mechanical conditioning but specialized hardware required
[79]	Human cardiac organoids containing CMs, endothelial/perivascular, fibroblast and epicardial populations	Vascularized multicellular cardiac organoids	Structural/functional: vascular cells increased maturation and contractile force; endothelial LAMA5 promoted mature sarcomeric protein expression; PDGF signaling increased matrix deposition and force	Higher biological complexity and dependence on multicellular organization	High physiological relevance; moderate scalability: strong disease-modeling potential
[80]	hiPSC-derived cardiac spheroids/organoids	Porous 3D microwell + suction-based large-scale production	Functional/organizational: improved homogeneity, functionality and sphericity; generated ~40,000 hiPSC cardiac spheroids at once; atrial/ventricular regions could be spatially organized	Primarily optimized production/homogeneity; maturation characterization is less comprehensive than EHT studies	Very high scalability: particularly relevant for manufacturing and regenerative applications
[81]	hiPSC-CM ± hiPSC-derived cardiac fibroblasts in EHT	Fibroblast–cardiomyocyte multicellular EHT	Structural/functional: fibroblast-containing EHTs showed ~5-fold higher normalized force and ~30% greater contraction amplitude than CM-only tissues	Limited maturation endpoints and small-scale EHT format	Limited scalability and small-scale EHT format.

As stated by Fick’s law of diffusion, in a passive system where diffusion is limiting, the diffusion time is directly proportional to the square of the distance [82]. In comparison to conventional well plates, microfluidic systems reduce characteristic diffusion distances and can additionally provide controlled convective transport [70].

## 4. Engineering 2D and 3D Platforms for Cardiac Differentiation

Cardiac engineering increasingly distinguishes stage-specific lineage specification from subsequent functional maturation, motivating the transition from conventional 2D cultures toward bioengineered 3D systems designed to reproduce native mechanical, biochemical, and multicellular cues.

### 4.1. Conventional 2D Systems

The evolution of human induced pluripotent stem cell (hiPSC) cardiac engineering highlights the fundamental distinction between early stage-specific lineage specification and subsequent functional maturation. Conventional two-dimensional (2D) planar cultures remain the primary workhorse for directing early fate specification due to their precise control over morphogen gradients and temporal β-catenin signaling modulation [7]. Monolayer differentiation protocols routinely yield high cardiomyocyte purity and efficiency, frequently exceeding 80–95% cTnT+ cells under chemically defined, serum-free conditions [83]. Furthermore, 2D monolayers afford direct single-cell optical access, enabling real-time high-resolution microscopy and patch-clamp electrophysiology to dissect cell-autonomous signaling cascades without the confounding variables of nutrient diffusion barriers. Despite these advantages, 2D monolayer systems present severe physiological failure modes that prevent complete functional maturation. Cells cultured on conventional plastic or glass substrates encounter extreme biomechanical mismatch, as substrate stiffness 1 GPa exceeds native myocardial elasticity (10–15 kPa) by several orders of magnitude. This non-compliant 2D environment deprives hiPSC-derived cardiomyocytes (hiPSC-CMs) of physiological mechanical feedback, locking them in an embryonic or fetal-like state characterized by disorganized, unaligned myofibrils, absent transverse tubules (T-tubules), depolarized resting membrane potentials (−50 to −60 mV due to low IK1 current density), and automaticity [84]. Crucially, hiPSC-CMs in 2D monolayers exhibit persistent metabolic immaturity, relying predominantly on glycolysis and lactate oxidation for ATP generation while possessing small, perinuclear mitochondria with well-developed cristae [15,54].

In contrast, adult cardiomyocytes derive 70–90% of their ATP from mitochondrial fatty acid β-oxidation. Resolving this metabolic bottleneck is of paramount clinical relevance because mitochondrial dysfunction and disrupted fatty acid oxidation are central hallmarks of myocardial ischemia, metabolic remodeling, and heart failure [51]. Consequently, the inability of planar 2D substrates to provide 3D cell–matrix interactions, dynamic strain, and multicellular crosstalk severely limits their capacity to model complex metabolic pathologies or predict clinical drug-induced cardiotoxicity in vitro.

### 4.2. Cardiac Spheroids

To overcome the physiological limitations of planar cultures, self-assembling three-dimensional (3D) cardiac spheroids (or cardiac microtissues) have emerged as a robust platform bridging conventional two-dimensional (2D) monolayers and complex tissue engineering constructs [85]. Spheroids spontaneously form through scaffold-free cell aggregation on ultra-low attachment surfaces, hanging-drop systems, or dynamic suspension bioreactors. This close spatial proximity promotes high-density cell–cell interactions—predominantly mediated by N-cadherin and connexin-43 (Cx43)—and stimulates the deposition of an endogenous extracellular matrix (ECM) rich in fibronectin and collagen type I/IV, which collectively complements stage-specific Wnt signaling by enhancing mesodermal priming, cell survival, and early structural patterning in human induced pluripotent stem cell (hiPSC) derivatives. A principal advantage of cardiac spheroids is their capacity to incorporate multiple relevant cardiac cell types in defined physiologically representative ratios. Multicellular co-culture spheroids combining hiPSC-derived cardiomyocytes (hiPSC-CMs) with human cardiac fibroblasts (hCFs), endothelial cells (ECs), and pericytes/smooth muscle cells closely emulate native myocardial paracrine signaling [50,77]. The presence of non-cardiomyocytes in these 3D microenvironments significantly drives maturation across all four major physiological domains [86].

(i) Structural maturation: heterotypic cell–cell contacts and paracrine signaling (e.g., VEGF, bFGF, and TGF-β) promote nascent transverse tubule (T-tubule) development, increased myofibrillar density, well-defined Z-discs, and localized intercalated disc assembly [86];
(ii) Electrophysiological maturation: enhanced Cx43 expression and intercellular coupling generate adult-like action potential profiles, hyperpolarized resting membrane potentials, and synchronized wave propagation [86];
(iii) Metabolic maturation: multicellular 3D architecture induces a notable metabolic transition, triggering the shift from embryonic glycolysis toward mitochondrial fatty acid β-oxidation, accompanied by increased mitochondrial mass, mature cristae morphology, and downregulation of HIF1α/LDHA-mediated glycolytic pathways [86];
(iv) Functional/mechanical maturation: constructs exhibit robust, synchronized spontaneous contractility, improved active force generation, and accelerated calcium-handling kinetics characterized by enhanced sarcoplasmic reticulum Ca^2+^ cycling via SERCA2a.

**Table 3:** Representative studies engineering the cardiac developmental niche for hiPSC-derived cardiomyocyte maturation.

Representative Study	ECM	Biochemical	Biomechanical	Bioelectrical	Cell–Cell Interaction	Microfluidics	Tissue Organization (2D/3D)	In Vivo Evaluation	Key Finding
[21]	✓	✓	✓	✓			✓ (2D)	✓	A biomimetic composite of Matrigel and fibronectin was engineered to provide a dual-modality ECM niche. By virtue of the strongest expression of cardiac-specific genes, this combination offers the most conducive ECM environment for promoting cardiac differentiation and maturation.
[61]		✓	✓	✓	✓	✓	✓ (3D)		A tri-culture heart-on-a-chip model using iPSCs, fibroblasts, and endothelial cells was reported. This model features thick myocardial tissue, strong spontaneous contraction and vascular endothelial cell layers, providing a significant advantage in being able to measure vascular permeability, which is closely related to the pathophysiology of atherosclerosis.
[22]	✓	✓	✓		✓		✓ (2D)		This study established that fibronectin is an indispensable, stage-specific driver of human precardiac mesoderm formation and survival.
[60]	✓		✓		✓	✓	✓ (3D)		Developed a perfusable microfluidic cardiac tissue platform incorporating decellularized heart extracellular matrix and multiple cardiovascular cell types, resulting in significantly enhanced structural and functional maturation and improved performance in drug screening, disease modeling, and regenerative applications.
[87]	**✓**		**✓**			**✓**	**✓** **(3D)**	**✓**	Established a micro-engineered heart-on-chip integrating hiPSC-derived cardiomyocytes with endothelial, smooth muscle, and fibroblast populations under continuous perfusion, promoting tissue self-organization, increased contractile performance, faster electrical conduction, and more physiologically relevant drug responses.
[88]	**✓**	**✓**		**✓**		**✓**	**✓** **(3D)**	**✓**	Reviewed engineering-driven cardiac organoid technologies demonstrating that combining extracellular matrix scaffolds, biochemical patterning, multicellular organization, electrophysiological assessment, and microfluidic platforms substantially improves organoid complexity and translational relevance for cardiovascular tissue engineering applications.
[7]	**✓**	**✓**					**✓** **(2D)**		Scalable, high-yield (>80–90% cTnT) differentiation via small-molecule Wnt modulation.
[86]	**✓**	**✓**			**✓**		**✓** **(3D)**		Multicellular crosstalk promotes T-tubule development and electrophysiological maturation
[89]	**✓**	**✓**	**✓**	**✓**	**✓**		**✓** **(3D)**		Progressive electrical training induces adult-like sarcomere length (~2.2 µm) and metabolic switching.

Note: **✓** indicates that the corresponding engineering cue was incorporated or evaluated in the cited study.

Furthermore, the integration of advanced bioelectronic interfaces into 3D tissue architectures represents a critical technological evolution for non-invasive, continuous functional monitoring. Paralleling developments in bioengineered neural platforms—such as lollipop-shaped spheroids equipped with integrated electrical recording technologies for high-fidelity electrophysiological tracking [90]—next-generation cardiac microtissues and organoids are increasingly incorporating flexible microelectrode arrays (MEAs) and bioelectronic scaffolds. These integrated platforms enable real-time, non-disruptive quantification of field potentials, action potential propagation, and electromechanical coupling in 3D cardiac constructs during long-term culture and cardiotoxicity screening.

Beyond conventional spheroids, recent bioengineering breakthroughs have driven the emergence of self-organizing cardiac organoids (“cardioids”) and microphysiological Heart-on-a-Chip (HoC) technologies. Unlike isotropic spheroids, cardiac cardioids recapitulate embryonic cardiogenesis through self-directed tissue patterning, forming hollow, chamber-like structures that mimic early heart-field development and cavity formation [79,88,91]. Stage-specific modulation of Wnt, BMP, and FGF pathways in hiPSC aggregates enables the generation of self-patterning cardioids capable of forming distinct chamber-specific compartments for modeling congenital heart defects in vitro. Concurrently, microfluidic HoC systems integrate dynamic physiological perfusion, providing continuous nutrient delivery, fluid shear stress, and multi-axial mechanical strain that prevent the formation of hypoxic necrotic cores while housing embedded sensors [92].

Despite these advanced physiological features and their high adaptability to high-throughput drug safety and toxicity testing in vitro [93], cardiac spheroids present intrinsic physical and biomechanical limitations. Due to the absence of a functional, perfused vascular network, spheroids exceeding 200–300 µm in diameter develop central hypoxic cores and steep nutrient/metabolite gradients that result in localized core necrosis. Furthermore, while spheroids provide superior cell–matrix and cell–cell contacts compared to 2D cultures, they lack the uniaxial mechanical alignment, dynamic cyclic strain, and bioelectrical pacing required to induce full anisotropic sarcomeric organization and adult-level contractile force. Consequently, spheroids serve as a crucial intermediate model, highlighting the necessity of integrating these 3D units into advanced bioengineered heart tissues and microfluidic platforms [93].

#### Cardiac Organoids and Heart-on-a-Chip Systems

Beyond conventional self-assembling spheroids, recent bioengineering breakthroughs have driven the emergence of self-organizing cardiac organoids (“cardioids”) and microphysiological Heart-on-a-Chip (HoC) technologies. Unlike isotropic spheroids, cardiac organoids recapitulate embryonic cardiogenesis through self-directed tissue patterning, forming hollow, chamber-like structures that mimic early heart-field development and cavity formation [79,88,91,94]. Recent studies demonstrate that stage-specific activation of Wnt, BMP, and FGF signaling cascades in hiPSC aggregates enables the generation of self-patterning cardioids capable of forming distinct atrial- and ventricular-like compartments, providing powerful models for studying congenital heart defects, cardiac looping, and chamber-specific pathologies in vitro [91,94].

Parallel advancements in microfluidic biofabrication have yielded sophisticated Heart-on-a-Chip (HoC) systems that integrate dynamic physiological variables absent in static cultures. By housing hiPSC-CMs and multicellular constructs within microfluidic channels, HoC platforms provide continuous vascular-like medium perfusion, preventing the formation of hypoxic necrotic cores typical of large spheroids while applying physiological fluid shear stress [92]. Furthermore, state-of-the-art HoC devices integrate multi-axial mechanical strain actuation (simulating ventricular afterload) alongside embedded microelectronic sensors, enabling real-time, label-free quantification of tissue impedance, action potential propagation, and contractile forces during chronic drug exposure [95,96]. These advanced microphysiological systems bridge the translational gap between simplified cell aggregates and native myocardial tissue, establishing robust tools for personalized cardiotoxicity testing and regenerative medicine [87].

### 4.3. Comparative Advantages of 2D and 3D Systems

Directly evaluating conventional 2D monolayers against bioengineered 3D platforms reveals a fundamental trade-off between experimental throughput and physiological fidelity [97]. Rather than viewing 2D and 3D systems as mutually exclusive, hiPSC-based cardiac culture platforms can be conceptualized across four distinct technological tiers across the drug discovery and tissue engineering pipeline:

2D Monolayer Cultures: Provide unmatched operational scalability, low assay cost, high batch-to-batch reproducibility, and direct single-cell optical access [7,98]. They serve as the primary engine for high-throughput small-molecule screening, early Wnt lineage specification sweeps, and cell-autonomous pathway dissection, despite their fetal-like structural and metabolic state [97,98].

Self-Patterning Cardioids/Chambered Organoids: Recapitulate early embryonic cardiogenesis through self-directed tissue patterning and hollow cavity formation [91]. They offer specialized utility for modeling congenital heart diseases, early cardiac morphogenesis, and developmental cardiotoxicity, though they lack uniaxial mechanical tension.

Complex Multicellular Heart Organoids: Integrate hiPSC-CMs with non-cardiomyocytes (hCFs, ECs, pericytes) in 3D cell aggregates [86]. Rich heterotypic paracrine crosstalk drives intermediate structural, electrophysiological, and metabolic maturation, serving as robust models for multicellular cardiac pathologies (e.g., cardiac fibrosis) and safety pharmacology [86,99].

Advanced Bioengineered Models (EHTs & Heart-on-a-Chip): Provide anisotropic alignment, uniaxial mechanical loading, bioelectrical pacing, and fluid perfusion [45,46,59]. These systems establish adult-like sarcomeric organization (2.2 μm), hyperpolarized resting potentials (−80 mV), and direct isometric force readouts (mN/mm^2^), serving as high-fidelity validation models for inotropic drug testing and preclinical remuscularization [100,101].

These biological enhancements in 3D systems translate into superior predictive accuracy during pharmacology and cardiotoxicity testing in vitro [102,103]. Whereas immature 2D hiPSC-CMs frequently produce false-positive or false-negative readouts regarding drug-induced proarrhythmia (e.g., hERG channel blockades) and inotropic responses, multicellular 3D microtissues can provide improved predictive performance for human-specific cardiotoxins (e.g., doxorubicin) and ion channel modulators [103]. However, 3D platforms face translational hurdles, including higher fabrication costs, technical complexity, lower throughput, hydrogel batch variability, and oxygen diffusion constraints (>200 μm) [97,102].

### 4.4. Engineered Heart Tissues

Engineered heart tissues (EHTs) represent an advanced tissue-engineering platform, integrating human induced pluripotent stem cell-derived cardiomyocytes (hiPSC-CMs) and supportive non-myocytes (such as human cardiac fibroblasts and endothelial cells) within three-dimensional hydrogel matrices—typically fibrin, collagen type I, or gelatin methacrylate—cast around flexible silicone (PDMS) posts, flexible pillars, or rigid frames. Unlike unconstrained spheroids, EHT platforms impose defined boundary conditions that generate passive mechanical tension along a longitudinal axis, promoting directional cell alignment, anisotropic electrical wave propagation, and dense sarcomeric organization [100].

The implementation of serum-free, chemically defined hydrogel protocols has enabled the generation of macro-scale engineered human myocardium with high structural homogeneity, predictable force generation, and tissue-level functional maturity suitable for preclinical cardiac repair and drug evaluation. To overcome the physiological immaturity typical of early-stage differentiation, advanced EHT platforms leverage a temporally staged application of microenvironmental cues: following initial Wnt-mediated lineage specification (Days 0–7) and 3D hydrogel encapsulation/self-assembly (Days 7–14), dynamic physical conditioning—combining bioelectrical pacing and auxotonic mechanical stretch—is initiated (Days 14+) [78,100,104]. Early biowire platforms demonstrated that controlled electrical pacing drastically enhances myofibrillar alignment, conduction velocity, and action potential kinetics compared to unstimulated controls [104]. Expanding on these principles, physical training regimes applying gradually increasing electrical pacing to early-stage cardiac tissues induce advanced adult-like maturation features within four weeks of culture [89].

(i) Structural maturation: achievement of physiological sarcomere lengths (~2.2 µm), well-developed transverse tubule (T-tubule) networks, aligned Z-discs, and mature intercalated disc assembly [89,104].
(ii) Electrophysiological maturation: hyperpolarized resting membrane potentials (~−80 mV), upregulated IK1 current density, fast action potential conduction velocities, and the elimination of immature automaticity [89,104].
(iii) Metabolic maturation: oxidative metabolic switching from fetal glycolysis to mitochondrial fatty acid β-oxidation, accompanied by a high mitochondrial volume fraction (~30% of cell volume) and cristae-dense ultrastructure [89].
(iv) Functional/mechanical maturation: development of direct isometric twitch force, positive force-frequency relationships (Bowditch staircase phenomenon), and mature calcium-handling kinetics mediated by sarcoplasmic reticulum SERCA2a and RyR2 cycling [89,101].

Beyond driving phenotypic maturation, EHTs provide an indispensable platform for direct, non-invasive quantification of contractile performance under physiological and pharmacological conditions in vitro [101]. Automated optical tracking of post deflection enables real-time measurement of twitch force, contraction/relaxation kinetics, and positive or negative inotropic responses to physiological regulators such as extracellular calcium and β-adrenergic agonists (e.g., isoprenaline). However, a critical synthesis of current EHT technology reveals significant technical and translational bottlenecks. EHT platforms suffer from low-to-medium throughput, elevated manufacturing costs, and substantial fabrication complexity compared to 2D cultures or 3D spheroids [100,101]. Furthermore, thick non-vascularized EHT constructs (>200–500 μm) face severe oxygen and nutrient diffusion limits that necessitate active microfluidic perfusion, while reliance on animal-derived hydrogels introduces batch-to-batch variability and regulatory barriers for good manufacturing practice (GMP)-compliant clinical translation [100]. Overcoming these limitations through synthetic hydrogel chemistry, automated biofabrication, and integrated microvascular networks remains essential for translating EHTs into cardiac repair applications and scalable in vitro toxicity models.

## 5. Future Perspectives

Although substantial progress has been achieved in directing human induced pluripotent stem cell (hiPSC) differentiation toward the cardiac lineage, generating cardiomyocytes that faithfully recapitulate the structural, electrophysiological, metabolic, and contractile properties of the adult myocardium remains one of the major challenges in cardiovascular tissue engineering. Recent studies indicate that future advances are unlikely to arise from further optimization of individual differentiation factors alone, but rather from the coordinated integration of multiple components of the cardiac developmental microenvironment. Increasing evidence demonstrates that biochemical, biomechanical, bioelectrical, metabolic, and cellular signals act synergistically during cardiac development, suggesting that recreating this complexity through bioengineering strategies will be essential for achieving robust and reproducible cardiomyocyte maturation [89,100]. Consequently, near-term research should prioritize the development of integrated cardiac developmental niches and standardized maturation endpoints capable of reproducing the spatial and temporal coordination of developmental cues rather than optimizing isolated signaling pathways.

The convergence of biomaterials science, tissue engineering, and microphysiological systems is expected to drive the next generation of hiPSC-based cardiac models by enabling the simultaneous control of multiple microenvironmental cues. Advanced platforms, including engineered heart tissues (EHTs), cardiac organoids, tunable hydrogels, three-dimensional bioprinting, and heart-on-chip technologies, provide opportunities to reproduce the structural organization and dynamic environment of the developing myocardium. Unlike conventional two-dimensional cultures, these systems enable the integration of extracellular matrix remodeling, mechanical loading, electrical stimulation, oxygen and nutrient gradients, and multicellular interactions within a physiologically relevant three-dimensional context [83]. However, their increasing complexity introduces challenges related to tissue heterogeneity, long-term stability, vascularization, process standardization, and scalability. Future work should therefore prioritize quantitative benchmarking across platforms to determine which combinations of microenvironmental cues provide reproducible gains in structural, electrophysiological, metabolic, and functional maturation rather than assuming that increased model complexity inherently results in improved physiological fidelity [46,105].

Beyond advances in biomaterials and tissue engineering, the integration of artificial intelligence (AI), machine learning (ML), and systems biology may contribute to the optimization of cardiac differentiation and maturation protocols. The increasing availability of transcriptomic, proteomic, metabolomic, and single-cell datasets provides opportunities to identify regulatory networks associated with cardiomyocyte lineage commitment and functional maturation. AI-driven approaches may assist in predicting differentiation outcomes, identifying candidate developmental regulators, and prioritizing combinations of biochemical and biophysical stimuli for experimental validation [84]. However, their broader applicability will depend on dataset size and quality, batch effects, cell-line variability, model interpretability, and the availability of standardized experimental endpoints. In addition, models trained using specific cell lines, protocols, or laboratory conditions will require independent experimental validation and assessment of transferability before they can support broadly applicable differentiation strategies [106].

Despite these technological advances, several critical challenges must be overcome before engineered hiPSC-derived cardiac tissues can be routinely translated into clinical and industrial applications. Variability among hiPSC lines, incomplete functional maturation, limited protocol reproducibility, manufacturing scalability, and compliance with good manufacturing practice (GMP) standards continue to represent major obstacles for widespread implementation. Future research should therefore prioritize standardized differentiation protocols, robust quality-control strategies, reproducible bioengineering platforms, and quantitative release criteria capable of generating clinically relevant cardiac tissues at scale. In the longer term, the convergence of developmental biology, stem cell science, bioengineering, and computational approaches may enable increasingly reproducible and physiologically relevant patient-specific cardiac tissues for precision medicine, high-throughput drug screening, disease modeling, and regenerative therapies [61,107].

## 6. Conclusions

Human induced pluripotent stem cells (hiPSCs) have transformed cardiovascular research by providing an accessible and versatile platform for studying cardiac development, disease mechanisms, drug discovery, and regenerative medicine. Although canonical Wnt signaling remains the cornerstone of cardiac lineage specification, efficient lineage commitment alone is insufficient to generate adult-like cardiomyocytes. As discussed throughout this review, structural, electrophysiological, metabolic, and functional maturation depends on the coordinated integration of biochemical, biomechanical, bioelectrical, metabolic, and cellular cues that collectively define the cardiac developmental microenvironment. Accordingly, engineering these complementary signals represents a key strategy for overcoming the immature phenotype that continues to limit the physiological relevance and translational utility of hiPSC-derived cardiomyocytes.

The evidence reviewed here supports a shift from optimizing cardiac differentiation primarily for cardiomyocyte yield toward designing integrated developmental microenvironments that promote reproducible functional maturation. However, important challenges remain, including variability among hiPSC lines, incomplete maturation, differences in maturation endpoints, limited protocol reproducibility, and the scalability and standardization of increasingly complex bioengineered systems. Thus, while engineered microenvironments and advanced cardiac platforms provide compelling opportunities to improve physiological relevance, their broader translational impact will depend on rigorous benchmarking, experimental validation, and manufacturing compatibility. In this context, moving beyond Wnt signaling should not be understood as replacing its central role in cardiac specification, but as embedding Wnt-driven differentiation within a controlled developmental niche capable of supporting more consistent and physiologically relevant human cardiac tissues.

## Figures and Tables

**Table 1:** Comparative spectrum and performance matrix of hiPSC-based cardiac culture platforms across structural, functional, and translational metrics.

Platform Tier	Key Structural & Functional Features	Key Advantages	Primary Limitations	Primary Applications
2D Monolayers	Planar single-cell sheets on rigid plastic/glass	High throughput, low cost, excellent optical access, single-cell electrophysiology	Rigid mechanics (>1 GPa), fetal phenotype, no 3D ECM or strain	High-throughput sweeps, lineage specification, cell-autonomous pathways
Cardioids	Self-organizing 3D structures with hollow cavities	Mimics embryonic chamber formation & cardiogenesis patterning	Lack of mechanical stretch, variable cavity size, non-aligned fibers	Congenital heart defects, cardiac morphogenesis, developmental toxicity
Complex Heart Organoids	Multicellular co-cultures (CMs, FBs, ECs, pericytes)	Rich heterotypic paracrine signaling, improved T-tubule & metabolic maturation	Isotropic orientation, no direct force readout, hypoxic core (>200 μm)	Multicellular pathology (fibrosis), cell crosstalk, drug safety screening
Advanced Bioengineered Models (EHTs/MPS)	Anisotropic hydrogels under mechanical stretch & pacing	Adult-like alignment, hyperpolarized Vm, direct active force (mN/mm^2^)	Low-medium throughput, high complexity, microfluidic requirement	Quantitative force kinetics, inotropic drug testing, heart failure modeling, cardiac patches

Note: hiPSC-CM: human induced pluripotent stem cell-derived cardiomyocyte, hCF: human cardiac fibroblast, EC: endothelial cell, EHT: engineered heart tissue, MPS: microphysiological system/Heart-on-a-Chip, CHD: congenital heart disease, ECM: extracellular matrix, T-tubules: transverse tubules.

## Data Availability

No new data were created or analyzed in this study. Data sharing is not applicable to this article.

## Abbreviations

The following abbreviations are used in this manuscript:

AI	Artificial Intelligence
CDM3	Chemically Defined Medium 3
cTnT	Cardiac Troponin T
CF	Cardiac Fibroblast
Cx43	Connexin-43
dECM	Decellularized Extracellular Matrix
EC	Endothelial Cell
ECM	Extracellular Matrix
EHT	Engineered Heart Tissue
GMP	Good Manufacturing Practice
GSK-3	Glycogen Synthase Kinase-3
hCF	Human Cardiac Fibroblast
hESC	CMs–human embryonic stem cell-derived cardiomyocyte
hiPSC	Human induced pluripotent stem cell
hiPSC-CM	hiPSC-derived cardiomyocyte
hPSC	Human Pluripotent Stem Cell
HUVEC	Human Umbilical Vein Endothelial Cell
HoC	Heart-on-a-Chip
IK1	Inward Rectifier Potassium Current
MEA	Microelectrode Array
MEAs	Microelectrode Arrays
ML	Machine Learning
PDMS	Polydimethylsiloxane
SMC	Smooth Muscle Cell
Wnt	Wingless-related integration site signaling pathway
